# PP55 Health Technology Assessment Scoping Definition For Health Regulation

**DOI:** 10.1017/S0266462324002265

**Published:** 2025-01-07

**Authors:** Margarete Oliveira, Maira Ramos, Erika Camargo, Erica Silva, Flávia Tavares Elias

## Abstract

**Introduction:**

The first phase of a health technology assessment (HTA) is to define the scope of the assessment and choose the appropriate type of studies. The HTA in Regulation has been produced in cooperation with the Oswaldo Cruz Foundation (Brasilia) and the Brazilian Health Regulatory Agency (Anvisa). This work reports the Brazilian experience with HTA priority-setting within the time period 2022 to 2023.

**Methods:**

Focusing on Anvisa’s 2022 to 2030 research themes, a workgroup developed a priority-setting cycle addressing the following four steps: (i) survey application to define problems and topics (August to October 2022); (ii) data compilation and exploratory searches by topic; (iii) workshops with technical areas (March to June 2023), aiming to refine themes, define research questions, and determine what types of studies will be conducted); and (iv) presentation of prioritized themes.

**Results:**

Forty-six topics at the intersection of HTA and health regulation were prioritized. Macro-agendas for medical devices and medicines were the most frequent. The studies (39 scoping reviews, nine rapid HTAs, and four technical reports) aimed at post-market surveillance, definition of regulatory problems, selection of the best regulatory options, and identification of the legal basis for the selected problem. These studies will be used to assess the impact of the proposed solutions on stakeholders and identify any potential risks associated with them. Lastly, the studies will provide recommendations for decision-making regarding health regulatory measures.

**Conclusions:**

By establishing technical cooperation between stakeholders to gather the best available evidence for regulatory decision-making processes through HTA studies, scoping definition based on the real needs of decision-makers has taken place. In this collaboration, experts were mobilized to expand response capacity and technical-scientific knowledge to support decision-making in health regulation.

